# Everybody's Page

**Published:** 1890-03-01

**Authors:** 


### EVERYBODY'S PAGE.

THE petition praying for permission to be granted to women to study medicine in Germany is said to have been flatly refused by Prussia, Wurtemberg, Saxony, and the Duchies of Hesse-Darmstadt and Saxe-Weimar. Noble, large-minded Germany !

A MEETING was held in Paris on the 2nd of this month to consider the question of cheap dwellings, and the success of the London workmen's dwellings was mentioned as being most encouraging. We are a long way off yet from what we hope one day to see as the condition of the homes of our working classes.

DR. KIDERA YASUATSU has come to the conclusion that the habitual stoop of the Japanese is due to their extreme politeness in always using the bent posture, which is considered deferential. There are people who think that our habit of constantly raising the hat in the streets causes colds, neuralgia, and a host of other miseries, so anybody wishing to be a benefactor to mankind might invent some new methods of politeness which would not inflict bodily ills.

A CURIOUS old privilege which was brought to light during the hearing of the Spitalfields Market arbitration case shows that the people of Enfield were granted a charter which made them "docket free," that is, exempt from all market and bridge tolls, in the time of the Great Plague, in recognition of their courage in supplying London with vegetables whilst the pestilence was raging. Mr. Thirgood, who had been formerly a market gardener attending Spitalfields Market, stated that until the market came into the hands of the present leaseholder he had never paid market tolls.

DR. RYAN, at one of the Australian Health Society's lectures, held forth on "Health, and How to Keep it," and beginning with infants, he said that for the maintenance of health in childhood, pure air, proper food, sleep, and suitable clothing were the essentials. And the word clothing reminds us to ask why do we see so many unfortunate little bits of humanity buttoned up in such excessively tight garments, tight gaiters, and tighter coats ? Perhaps it is the fashion ! A most unfortunate one. It would be well if kind folk would only realize that little children are just like plants, and want plenty of room to grow in, and that they would develop much stronger limbs and stouter chests if they were allowed many extra inches of stuff in fewer and more sensible garments.

AT last somebody is taking up our wild birds' cause again, and the Rev. F. O. Morris, the ornithologist, is doing his best to send a petition to Parliament on their behalf this coming session. The Wild Birds' Protection Act did something, but Mr. Morris wants to have bird-nesting made punishable excepting during a certain limited time, for which time licences will be issued. Many wild birds are becoming rarer yearly owing to the raids which small boys and collectors make on the nests in the spring time, and parents, who ought to know better, encourage it by allowing their children to go nesting day after day under the pretence of making an egg collection. Sometimes, no doubt, birds do increase in such numbers as to need occasional destruction, as has been the case lately in Northamptonshire with the sparrows which had become a regular pest, but on the other hand, in pointing out and making the most of the bad they do, people are apt to forget the immense amount of good done by insectivorous birds.

DR. FREDRIK BJÖRNSTROM, physician of the Stockholm Hospital, who has written a book called "Hypnotism : Its History and Present Development," is of opinion that hypnotism should be dealt with by the Legislature like deadly poisons, and none but regular physicians allowed to practise it.

MR. TREVES, in one of his published lectures, relates a very good story about a girl who went to his out-patient rooms at the London Hospital, very lame. He told her he must amputate one of her toes, and the girl was quite pleased, because she thought the operation would enable her to wear a still smaller shoe !

IN this country a quack would not find many patients if he contented himself merely with asserting that on the average he cured more than he killed. To be successful his nostrum must be an infallible remedy for every complaint under the sun. Not so, however, in Mexico, where a practitioner of two years' standing, provided he can show more cures than kills during that period, holds the position of a recognised medical man, regardless of whether he has won a diploma or not !

PROFESSOR STUART has given some very interesting specimens of British crabs to the museum of the Royal College of Surgeons. These crabs, being desirous of prolonging their existence, and anxious to avoid being gobbled up or otherwise ill-treated by their numerous predatory enemies, detach bits of seaweed and zoophites with their nippers, and cover the exposed parts of their bodies up in them. As this habit is continued when the crabs are blind, it is not due, as some unkind people might suppose, to vanity, but instinct teaches them to copy their surroundings, and thus avoid detection.

MEN are willing to be scared a little, but not too much, and last week's *Medical Record* points out that sanitarians, in their anxiety for the public welfare, are apt to become alarmists. Laws for sanitation are, of course, absolutely necessary, but in order to procure the support of the people, their intelligence and common sense must be appealed to. The public generally is willing to put up with a great deal for the good of the community, but their patience and faith have limits, and when men are forbidden certain things in which their forefathers indulged and managed to live to a good old age on the ground that they contain the "germs of disease," they begin to doubt and think that the false alarms exist only in the mind of the over-conscious sanitarian.

AT the meeting of the Church of England Funeral Reform Association this month evidence was shown that at the Tower Hamlets Cemetery there were about 247,000 bodies crowded into a small area, and in one grave the bodies of 81 children were crammed, and then people who object to cremation talk about respect being shown to their dead ! And matters are not likely to improve with the increase of population and building. In "Cremation and Urn Burial," a section of a portion of a South London cemetery is given where there are 14 coffins one above the other to within four feet of the surface. The cemetery is being surrounded by buildings until very soon it will be quite as much in the midst of houses as any of the old yards which have been closed. If the general public could once realise the danger of this overcrowding they would be less given to abusing cremation. There are those who object to it from religious scruples, and say that it is against the doctrine of the Resurrection, and for them the late Lord Shaftesbury's answer is the best—"What then will become of the blessed martyrs, who in ancient and modern persecutions, have died at the stake ?"

